# T790M mutant copy number quantified via ddPCR predicts outcome after osimertinib treatment in lung cancer

**DOI:** 10.18632/oncotarget.25332

**Published:** 2018-06-15

**Authors:** Jacky Yu-Chung Li, James Chung-Man Ho, Kam-Hung Wong

**Affiliations:** ^1^ Department of Clinical Oncology, Queen Elizabeth Hospital, Hong Kong SAR; ^2^ Division of Respiratory Medicine, Department of Medicine, The University of Hong Kong, Queen Mary Hospital, Hong Kong SAR

**Keywords:** adenocarcinoma, lung carcinoma, osimertinib, ddPCR, mutant copy number

## Abstract

Osimertinib prolongs progression-free survival (PFS) in patients with metastatic, epidermal growth factor receptor (EGFR) T790M-mutated, non-small cell lung cancer (NSCLC) after failure of EGFR tyrosine kinase inhibitor (TKI) therapy. We investigated the utility of T790M mutant copy number quantification in a plasma cell-free DNA (cfDNA) assay for predicting clinical outcomes of osimertinib treatment. We retrospectively examined 161 patients who underwent plasma EGFR testing using a digital droplet polymerase chain reaction (ddPCR) technique after EGFR-TKI failure. Of the 74 (46%) patients with detectable T790M mutations in plasma, 55 received osimertinib treatment. Patients who achieved partial response had a higher plasma mutant copy levels than those with progressive disease. Patients who achieved stable disease also tended to have higher plasma mutant copy levels than those with progressive disease. High mutant copy number (≥ 105 per mL of plasma) was associated with shorter PFS (median: 5.5 months vs. not reached) and overall survival (median: 9.1 months vs. NR). Quantitative measurements of T790M mutant copy number in plasma cfDNA by ddPCR thus predicted treatment response and survival outcomes after osimertinib in NSCLC patients resistant to EGFR TKI.

## INTRODUCTION

Advancements in the understanding and detection of genomic changes in lung adenocarcinoma have the potential to greatly improve therapies for lung cancer patients. In the Asia-Pacific region, EGFR mutations are the most common mutation in patients with lung adenocarcinoma, and are present in around 30% of patients with a history of smoking and 60% of patients who never smoked [1]. Specifically, a deletion in exon 19 or an L858R point mutation in exon 21 identified via tissue biopsy account for over 90% of all known sensitizing mutations and are associated with good responses to first- and second-generation EGFR TKIs. Unfortunately, half of these patients develop resistance to EGFR TKIs within 9-12 months [2–6]. The exon 20 T790M mutation is the primary cause of this resistance, and inhibitors that specifically target T790M have been developed. Osimertinib, a specific T790M inhibitor that was fast-track approved by the US FDA in November 2015 for treatment of patients with metastatic EGFR-T790M mutation-positive NSCLC as detected by an FDA-approved test (Cobas^®^ EGFR Mutation Test v2) after EGFR-TKI failure, achieved overall response rates (ORR) of up to 70% and progression-free survival (PFS) times of around 12 months [7]. However, clinical detection of the EGFR-T790M mutation through tissue biopsy can be challenging due to risks associated with the biopsy procedure and because of spatial and temporal tumor heterogeneity. Methods for detecting the T790M mutation using liquid biopsies may be useful as less-invasive, preliminary screening methods; tissue biopsies would then be required only for patients with negative liquid biopsy results [8].

Liquid biopsy methods for detecting T790M in cell-free (cf) DNA can be qualitative or quantitative, and quantitative assays generally have much higher sensitivity [9]. Currently available quantitative detection methods for evaluating EGFR mutation status include BEAMing (beads, emulsion, amplification, and magnetics), digital droplet polymerase chain reaction (ddPCR), and next generation sequencing (NGS)-based methods. Each of these approaches has its own advantages and disadvantages [10–13]. Here, we used a commercially available ddPCR kit for clinical use because it was the least expensive method and had the shortest turnaround time at 48 hours.

In this study, we investigated the usefulness of ddPCR for quantitative detection of EGFR mutations in plasma samples and for guiding decisions regarding osimertinib therapy in lung cancer patients in a community setting who had developed resistance to first- or second-generation EGFR-TKI.

## RESULTS

### Patient population

A total of 161 patients were enrolled in cohort A. T790M mutations were detected via ddPCR in 74 of these patients, but only 55 received osimertinib and were enrolled in cohort B. Nineteen patients with detectable T790M mutations were not treated with osimertinib due to rapidly fatal disease (n=18) or extreme renal impairment (CrCl < 10 mL/min) (n=1). Patient characteristics for each cohort are listed in Table 1.

**Table 1:** Patient's characteristics of study cohorts A and B

	Cohort A (Progression group) (N= 161)	Cohort B (Osimertinib group) (N=55)	P-value
Age – year			
Median (range)	66 (40 - 96)	65 (40 - 89)	0.12
Sex – no. (%)			
Male	70 (43.5%)	24 (43.6%)	0.98
Female	91 (56.5%)	31 (56.4%)	
Ethnic – no. (%)			
Asian	161 (100.0%)	55 (100.0%)	0.43
Non-Asian	0 (0.0%)	0 (0.0%)	
Histologic type – no. (%)			
Adenocarcinoma	153 (95.0%)	52 (94.5%)	0.87
Adenosquamous carcinoma	4 (2.5%)	2 (3.6%)	
Other	4 (2.5%)	1 (1.8%)	
Smokers – no. (%)			
Never	128 (79.5%)	46 (83.6%)	0.65
Former	26 (16.1%)	8 (14.5%)	
Current	7 (4.4%)	1 (1.8%)	
M-staging – (%)			
M0	7 (4.4%)	0 (0%)	0.65
M1a	48 (29.8%)	16 (29.1%)	
M1b	106 (65.8%)	39 (70.9%)	
Known EGFR mutation type – no. (%)			
Exon 19	82 (50.9%)	35 (63.6%)	0.22
L858R	68 (42.2%)	16 (29.1%)	
Others	11 (6.8%)	4 (7.3%)	
No. of prior EGFR TKI ever received – no. (%)			
1	157 (97.5%)	52 (94.5%)	0.28
2 or more	4 (2.5%)	3 (5.5%)	
No. of prior chemotherapy ever received – no. (%)			
0	119 (73.9%)	33 (60%)	0.15
1	31 (19.3%)	16 (29.1%)	
2 or more	11 (6.8%)	6 (10.9%)	
ECOG – no. (%)			
0-1	118 (73.3%)	2 (76.4%)	0.71
2	32 (19.9%)	8 (14.5%)	
3	10 (6.2%)	4 (7.3%)	
4	1 (0.6%)	1 (1.8%)	

### Cohort A – diagnostic analysis

**Figure 1:**
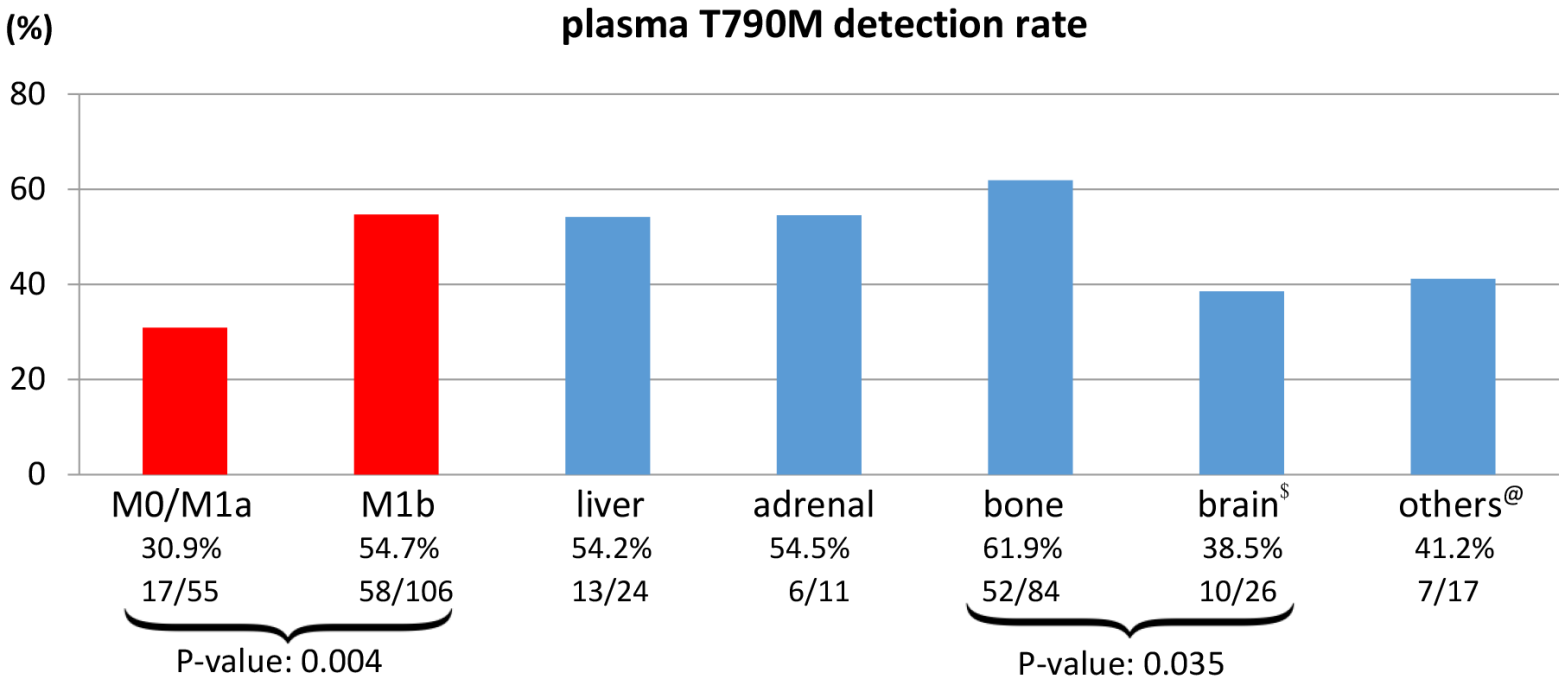
Plasma T790M detection rates in M0, M1a, M1b stage and M1b sub-site (liver, adrenal, bone, and others) patient subgroups ^$^Brain includes brain parenchymal and leptomeningeal metastases. ^@^Others includes skin and soft tissue metastases. Reduced cfDNA release in patients with brain metastases explains the lower detection rate.

The plasma T790M mutation was detected in 74 out of 161 (46.0%) patients who experienced disease progression after first- or second-generation EGFR-TKI therapy. The median mutant copy number was 56.5 mutant copies/mL plasma (range: 3.5 to 65,887.3) at the time of radiological disease progression. All except four patients (median: 104 days, range: 75 to 124 days) who experienced progression had previously received EGFR TKI treatment for more than five months (median: 473 days, range: 170 to 2213 days). We then explored associations between detection of a T790M mutation and M-stage status (M0, M1a, M1b, and different M1b sub-sites) as determined by the TNM classification 7^th^ edition. The detection rate was higher in patients with M1b disease than in those with M0/1a disease (54.7% vs 30.9%, *p*=0.004); detection rates for patients with different M1b sub-sites are depicted in Figure 1. The detection rate was highest in patients who had bone metastases (61.9%) and lowest in those with brain metastases (38.5%).

A majority of the patients in this study either declined or were not considered candidates for tumor rebiopsy. Thirteen out of 87 (15%) patients with negative T790M results from plasma testing proceeded to tissue biopsy; T790M mutations were detected in only three of these patients. Both tissue rebiopsy and plasma EGFR testing were performed in only 16 (9.9%) patients (Table 2). Patients with positive T790M results from plasma testing generally did not undergo a tissue biopsy before osimertinib treatment in accordance with our institutional practice. The small patient sample size and lack of tissue rebiopsy confirmation of plasma T790M detection precluded meaningful evaluation of the sensitivity and specificity of the ddPCR assay, which was beyond the scope of this study.

**Table 2:** Correlation of tissue and plasma EGFR testing in cohort A

	Detectable T790M mutant on plasma (n=3)	No detectable T790M mutant on plasma (n=13)
Detectable T790M mutant on tissue rebiopsy (n=3)	0	3
No detectable T790M mutant on tissue rebiopsy (n=13)	3	10

### Cohort B – diagnostic analysis

**Figure 2:**
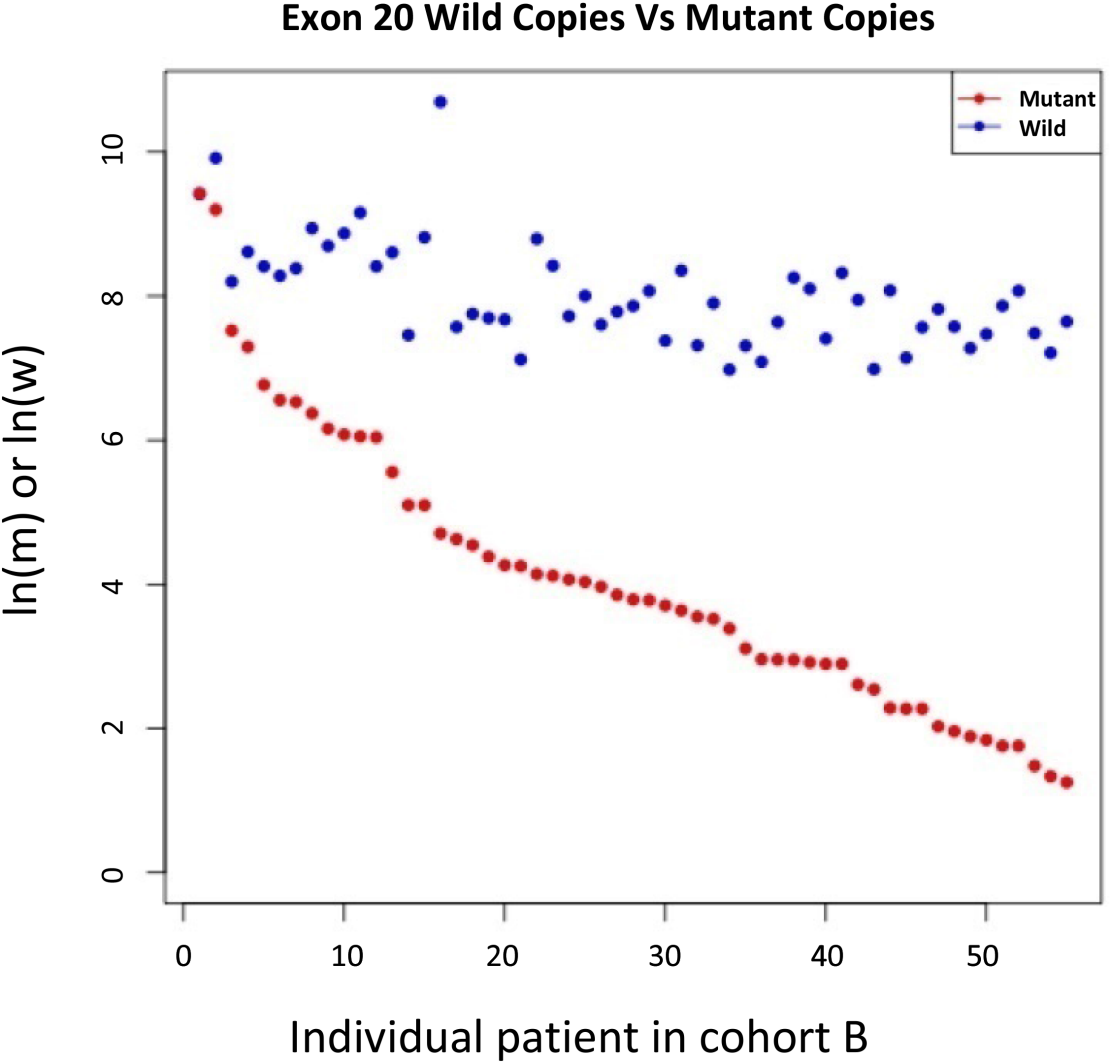
ln(mutant copy number/mL plasma) and ln(wild type copy number/mL plasma) for samples from all Cohort B patient Wild-type copy numbers were relatively more stable than mutant copy numbers in the majority of these patients.

The natural logarithms of T790M mutant copy number/mL plasma (ln(m)) for samples from the 55 patients who received osimertinib were arranged in descending order and plotted on a single graph with the corresponding natural logarithm of wild-type (exon 20) copy number/mL plasma (ln(w)) (Figure 2). Although wild-type copy number appeared to be relatively stable across the samples, it was moderately correlated with mutant copy number (Pearson correlation coefficient: 0.649).

### Cohort B – clinical outcomes analysis

**Figure 3:**
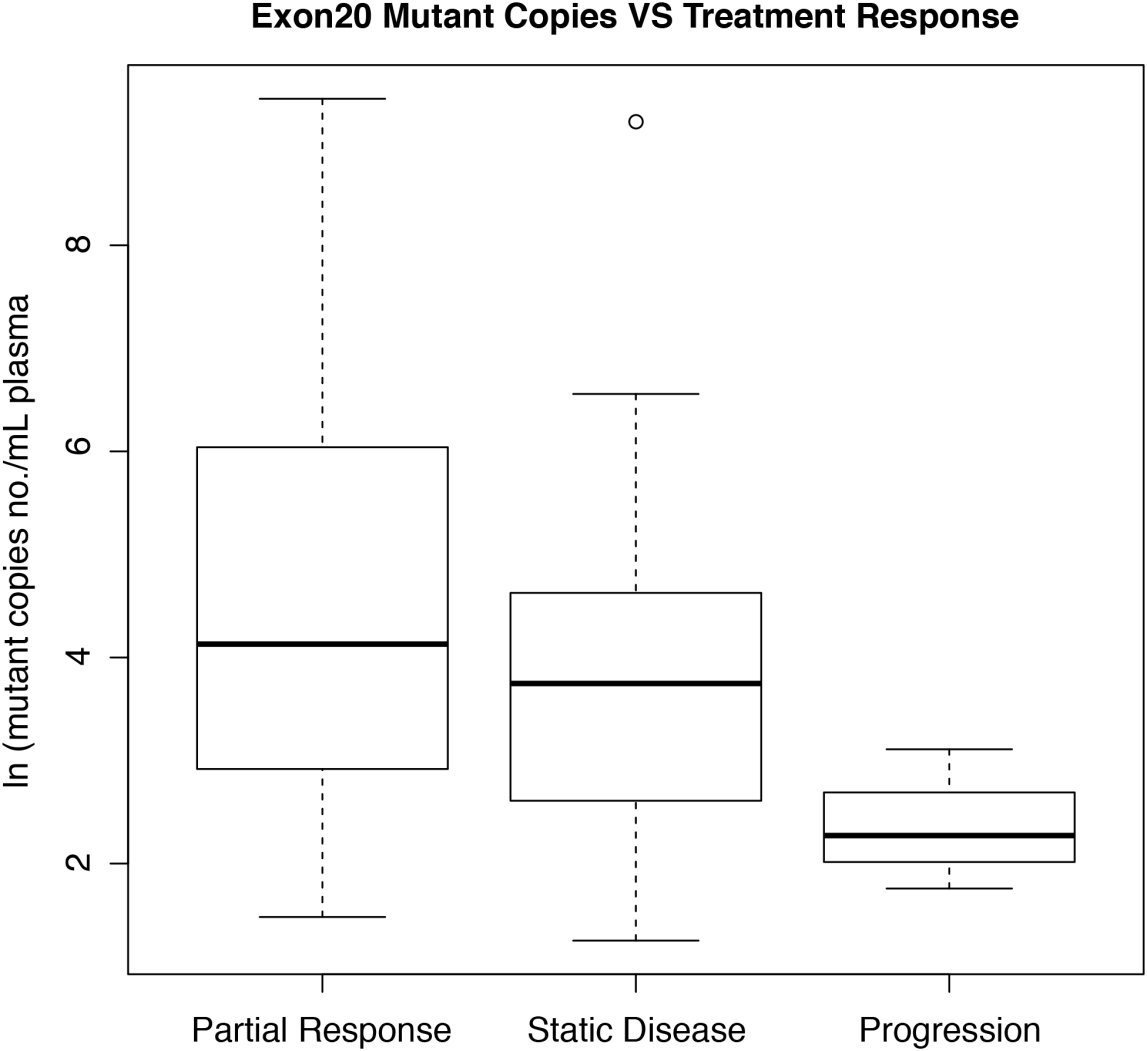
Box and whisker plot of correlations between ln(mutant copy number/mL plasma) and best treatment response The central box encompasses the 25^th^ and 75^th^ percentiles. Note: One data point in the SD group is located outside 1.5 times the interquartile range above the upper quartile and below the lower quartile.

Among the 55 patients who were treated with osimertinib, 35 (63.6%) achieved a partial response, 17 (30.9%) had stable disease, and only 3 (5.5%) had progressive disease as their best ORR. Natural logarithm of mutant copy number/mL plasma (ln (m)) values for the PR, SD, and PD subgroups are depicted in Figure 3. ln(m) was higher in the PR subgroup than the PD subgroup (mean ± SD: 4.33 ± 1.91 vs. 2.38 ± 1.50; *p*=0.031). However, there was also a trend towards higher ln(m) values in the SD subgroup than the PD subgroup (mean ± SD: 3.56 ± 0.682 vs 2.38 ± 1.50; *p*=0.07). ln(m) values did not differ between the PR and SD subgroups (*p*=0.16). Similar results were obtained when mutant percentages were compared among treatment response subgroups instead of ln(m).

**Figure 4:**
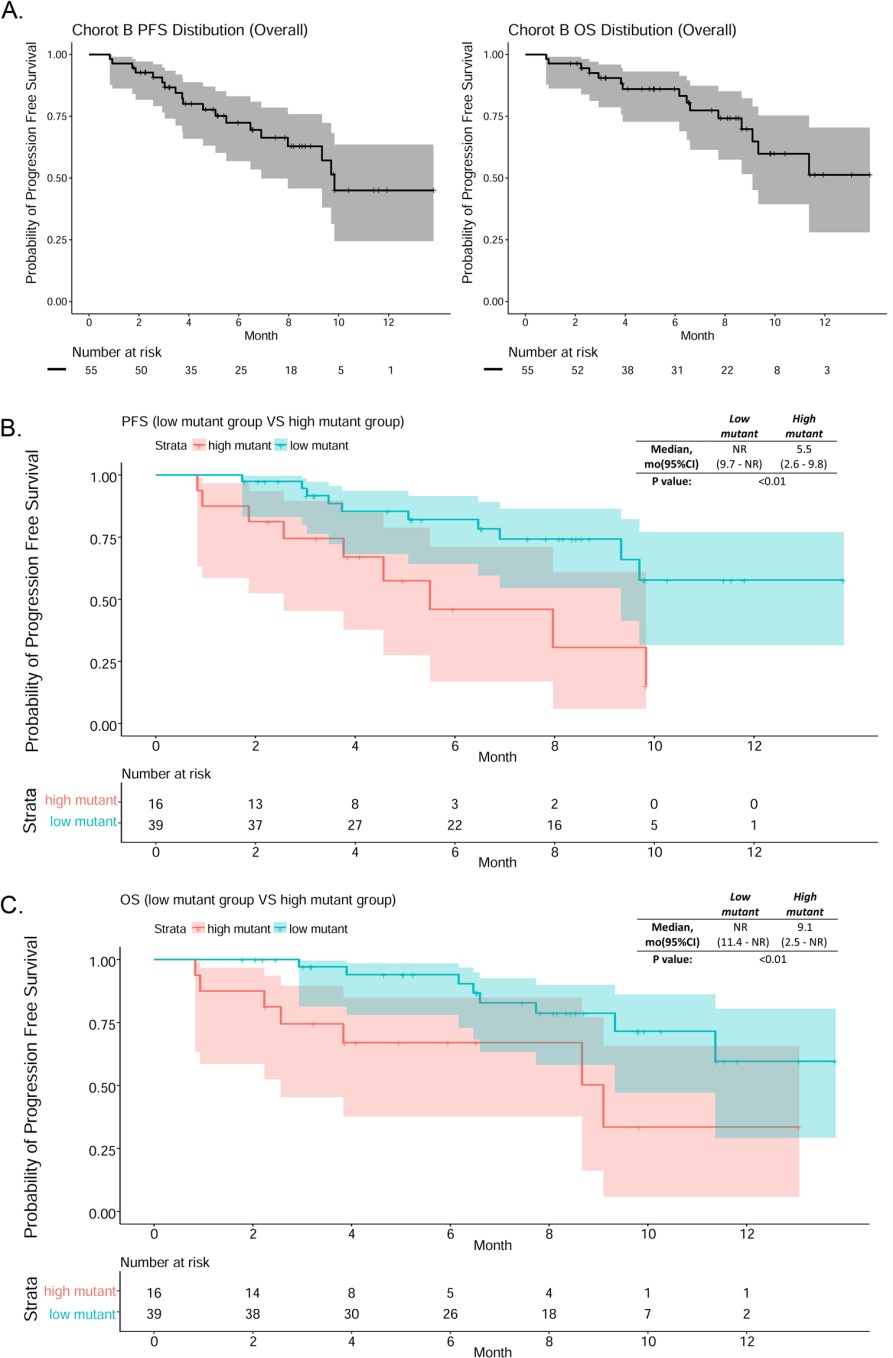
**(A)** PFS and OS curves^#^ of cohort B patients. **(B)** PFS curves^#^ for the low and high mutant copy number patient groups. **(C)** OS curves^#^ for the low and high mutant copy number patient groups. #Shaded areas (gray or color) are the 95% confidence intervals for each time point.

At the time when data collection ended, the median follow-up time was 8.4 months and median PFS was 9.8 months; median OS could not be determined. Survival curves are illustrated in Figure 4A.

A regression method was used to determine the cut-off value of T790M mutant copy number/mL plasma by categorizing Cohort B patients into high and low mutant groups using all cutoff values between 0 to 300 in increments of 5. Kaplan-Meier curves were generated for each potential cut-off value and the *p*-value for the difference between the high and low groups was calculated. The lowest *p*-value was obtained when 105 mutant copies/mL plasma was used as the cut-off; this value was therefore used to classify patients as having high or low plasma mutant copy numbers in subsequent analysis. The PFS and OS curves for the high and low mutant subgroups are shown in Figure 4B and 4C. Median PFS (5.5 vs NR, *p* < 0.01) and median OS (9.1 vs NR, *p* < 0.01) were shorter in the high mutant group than in the low mutant group. The high and low mutant groups did not differ with respect to age (*p*=0.223), sex (*p*=0.557), mutation type (del19 or L858R) (*p*=0.957), M-stage (M0/1a/1b) (*p*=0.827), or post-PD treatment (supportive care or chemotherapy) (*p*=0.308).

## DISCUSSION

Osimertinib received accelerated approval from the US FDA for treatment of patients with metastatic EGFR T790M mutation-positive NSCLC in November 2015. It received regular FDA approval following data consolidation and publication of the AURA3 study, which showed that osimertinib improved median PFS by 5.7 months (10.1 months vs 4.4 months, hazard ratio: 0.30) over platinum-pemetrexed in EGFR T790M-positive lung cancer patients with disease progression after first-line EGFR-TKI therapy [14]. In this retrospective study, we demonstrated the clinical efficacy of ddPCR for detecting T790M mutations in plasma samples from patients with acquired EGFR-TKI resistance and explored the ability of mutant copy number quantification via ddPCR to predict treatment outcomes.

The landmark AURA phase 1 and 2 studies revealed that osimertinib treatment was similarly effective in patients with T790M mutations whether they were identified by a plasma assay or by a tissue-based assay. Approximately 54% of EGFR-TKI resistant patients can be identified as T790M-positive by plasma using BEAMing; tissue rebiopsy might therefore be avoidable in these patients [8]. Similarly, in our study, tissue biopsies for identifying the T790M mutation were replaced by a ddPCR technique in 45% of patients. A cross-platform comparison study of various technologies for detecting T790M mutations demonstrated that the ddPCR and BEAMing digital platforms both had sensitivities of 71%, which substantially out-performed the non-digital Cobas and ARMS platforms with sensitivities of 29-41% [9]. Zheng *et al.* reported a similar cumulative positive detection rate of 44.6% at the time of radiological PD [15] when EGFR mutant copy levels were dynamically measured every two months before clinical evidence of PD. To the best of our knowledge, ours is the only community-based study to validate the usefulness of ddPCR instead of invasive tissue biopsies for detecting mutations in NSCLC patients with acquired resistance to EGFR-TKI.

Furthermore, we found that quantitative measurements of mutant allele copy numbers obtained via ddPCR predicted response to osimertinib treatment. A high T790M mutant allele copy number might be indicative of a higher percentage of cells in tumor lesions harboring the T790M mutation and of more severe T790M-dependent resistance. A rapid and dramatic decrease in mutant copy numbers upon treatment with a specific T790M inhibitor would therefore constitute a clinical treatment response. On the other hand, we found that PFS and OS were reduced in patients with high mutant copy numbers (above 105 copies/mL plasma). It is therefore possible that high mutant copy numbers reflect higher tumor loads and more aggressive tumors. Because later disease stages are intrinsically associated with poorer prognosis, and because of heterogeneity in resistance mechanisms within tumors [16], even exposure to a potent T790M inhibitor may not be able to completely reverse disease progression.

Since the EGFR T790M mutation was discovered in 2005 [17], extensive efforts have been made to identify associations between this mutation and clinical outcomes. However, conflicting results have been obtained regarding the impact of the T790M mutation on the development of acquired resistance. Some retrospective studies involving tumor re-biopsies indicated that NSCLC patients with acquired T790M mutations survived longer than those without these mutations [18–20]. On the other hand, Zheng *et al*. demonstrated in a prospective study that detection of T790M in plasma sample cfDNA from patients receiving TKI predicted poorer prognosis [15]. It seems that the prognostic implications of the T790M mutation may depend on the relative aggressiveness of the tumor and on the strategies used to overcome various non-T790M mechanisms of acquired resistance, including the use of etoposide-platinum for small cell transformation [21], anti-EGFR monoclonal antibody treatment, or a MET inhibitor in combination with EGFR-TKI [22, 23]. Unfortunately, except for one study that examined treatments after disease progression and TKI re-challenge [19], none of the aforementioned papers described the post-resistance treatments that patients received in detail. Regardless, the development of a specific T790M inhibitor has diminished the importance of T790M as a prognostic factor. Additional research is needed to identify prognostic and predictive factors in patients treated with osimertinib.

In this study, only 15% of patients ultimately underwent tissue rebiopsy following a negative liquid biopsy result. Previous studies have reported success rates ranging from 73% to 95% for tissue rebiopsies following negative liquid biopsies [24]. In clinical practice, invasive biopsy procedures are often difficult to arrange; in addition, many patients are reluctant to undergo the procedure, and poor patient performance status (26.7% in Cohort A had ECOG ≥ 2 in this study) and lack of awareness on the part of treating physicians might have also played a role. Furthermore, the sensitivity of plasma cfDNA testing is higher in patients with extra-thoracic metastases, multiple metastatic sites, or with hepatic or bone metastases; in general, these factors reflect a higher overall tumor burden [9, 25, 26]. The higher detection rates observed here in selected subgroups (M1b, bone, adrenal, and liver metastases) agree with previous reports. In those patients, the treating physician was less likely to order a confirmatory tissue rebiopsy due to the decreased likelihood of false negative results from the liquid biopsy; that might partially account for the low rebiopsy rate in cohort A patients. Although tissue biopsy is still considered the gold standard, liquid biopsy methods are continuously improving and are becoming applicable to a much broader population. Reckamp *et al.* were the first to successfully obtain matched tissue, plasma, and urine genotyping data in the TIGER-X phase I/II trial; that study reported that combined urine and plasma liquid biopsies had higher detection sensitivity (96.6% vs. 83.3%) for T790M mutants than tissue biopsy, and objective response rates were similar among all liquid and tissue biopsy methods [27]. As less invasive liquid biopsy methods continue to improve, it is possible that they may replace tissue biopsies as the preferred method for mutant detection.

Some limitations of this study should be considered when interpreting the results. This was a retrospective and single-institution study, and matched tissue and liquid biopsy results were not available for most of the patients. In addition, detection rate was used to indirectly reflect the sensitivity of the plasma EGFR testing in the M-stage and metastasis site subgroups. Finally, the institutional protocol for disease reassessment imaging was not highly standardized, which inevitably reduced the accuracy of treatment response and date of disease progression data. However, due to the consistent differences in OS and PFS in the cohort B subgroup analysis, we believe that the differences in PFS identified here are genuine.

## MATERIALS AND METHODS

### Patients

**Figure 5:**
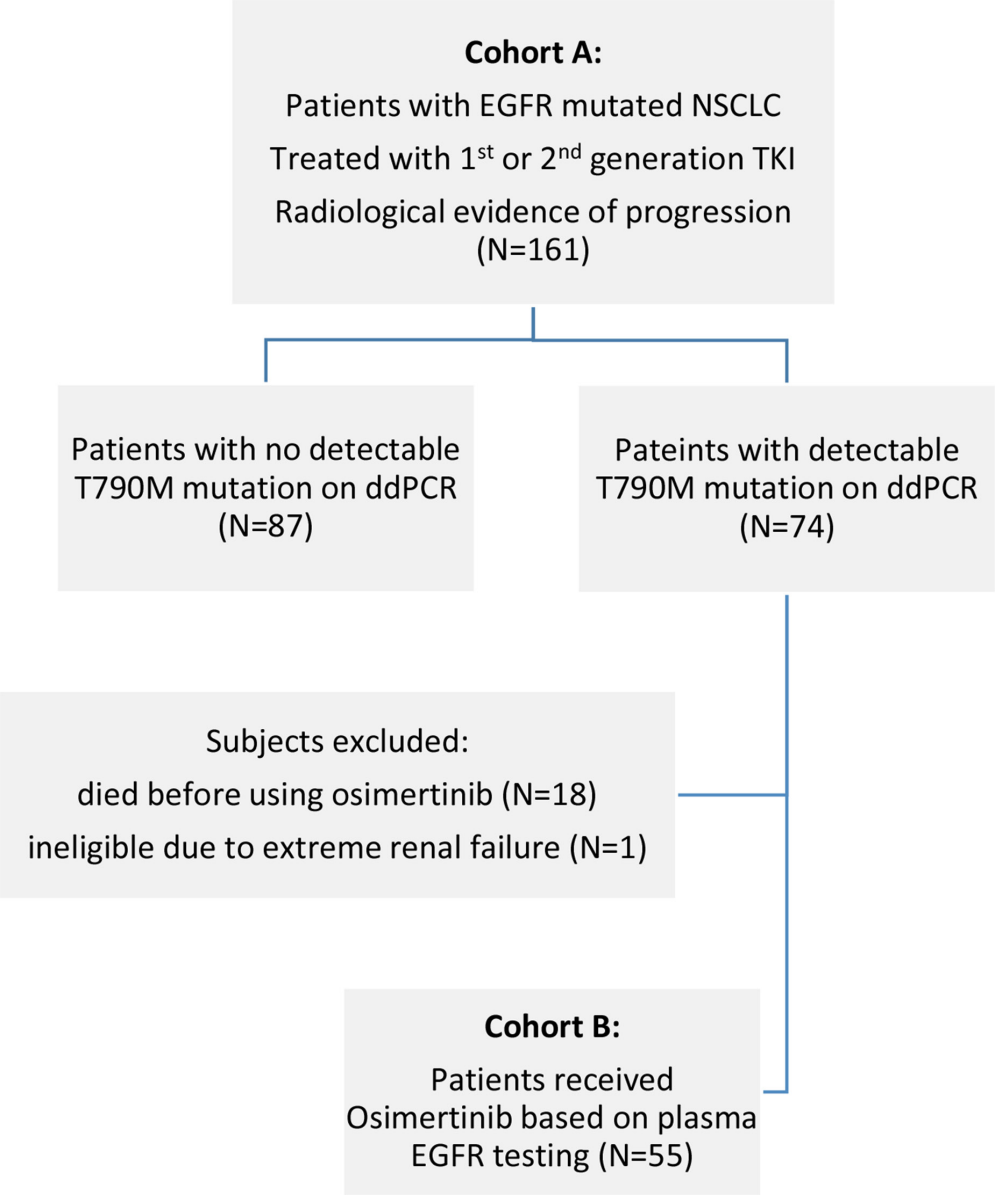
Flow diagram of study population for cohorts (A) and (B)

One hundred and sixty-one patients from the Department of Clinical Oncology, Queen Elizabeth Hospital, Hong Kong, who experienced progression after EGFR-TKI and had undergone plasma EGFR testing using the ddPCR technique between November 2015 and December 2016 were retrospectively identified and examined in this study. Since November 2015, all patients at our institution who developed resistance to EGFR-TKI were given the option of plasma EGFR testing that was followed by tissue re-biopsy in the event of a negative result. In addition to patients with a defined EGFR-TKI benefit per Jackman's criteria, plasma testing was also offered to non-responders who progressed within 6 months of EGFR TKI initiation. Patients in whom T790M mutations were identified received 80 mg of osimertinib orally every day until radiological disease progression or unacceptable toxicities occurred. The most recent staging computed tomography (CT) scan or positron emission tomography (PET)-CT scan in which radiological disease progression was observed after EGFR TKI served as the baseline. After osimertinib treatment began, tumors were reassessed every 4 to 6 weeks with chest x-rays and every 3 to 4 months with an appropriate imaging method, such as contrast CT scan, PET-CT, or magnetic resonance imaging of the brain, per institutional protocol. Radiological assessments were retrospectively reviewed in this study to evaluate responses to treatment. The following two patient cohorts were identified (Figure 5): Cohort A patients experienced disease progression during EGFR TKI treatment (progression group), and Cohort B patients received osimertinib based on ddPCR results (osimertinib group).

### Plasma EGFR testing

This study was approved by the institutional ethics committee of the hospital. All patients consented to plasma collection for cfDNA analysis to determine whether osimertinib treatment was recommended. The p-EGFR liquid biopsy mutation test by ddPCR for cfDNA analysis (Sanomics, Hong Kong) was used in this study. The same ddPCR protocol used in the ASPIRATION study [28], which was conducted using an X100 ddPCR machine, was used here on an updated X200 model ddPCR machine from BioRad. ddPCR was performed as follows. Ten to 20 mL of blood were collected in containers with EDTA and sent to the laboratory within 3 hours. Plasma was isolated from the whole blood via double-step centrifugation at 1,600g for 10 minutes and 16,000g for another 10 minutes at 4°C. DNA was extracted from plasma using the QIAamp DSP DNA Blood Mini Kit (Qiagen, Germany) and loaded into a QX200 Droplet Generator (BioRad, CA, USA) to generate water-in-oil droplets. Approximately 15,000-20,000 reaction droplets in which digital PCR reactions took place were generated in each well. These droplets were transferred into a 96-well plate and loaded onto a C1000 Touch™ Thermal Cycler (BioRad) where PCR amplification at the single molecule level took place within each droplet. Each droplet was then analyzed using a QX200 Droplet Reader (BioRad). QuantaSoft™ software, which utilizes Poisson statistics for absolute quantification of target DNA molecules, was used for data analysis. Numbers of mutant and wild-type DNA copies were calculated, and percent mutant values were calculated by dividing the number of mutant DNA copies by the total number of DNA copies in the plasma sample.

### Outcomes

All diagnostic scans were reviewed, and clinical efficacy outcomes were assessed in terms of objective response rate (ORR), PFS, and OS. PFS was defined as the time period from the first day of osimertinib treatment to the day of radiological evidence of disease progression. Best ORR was defined as the best radiological response observed in two successive imaging scans taken at least 4 weeks apart and on the basis of evaluations using Response Evaluation Criteria in Solid Tumors (RECIST) version 1.1. Demographic information and mutant allele quantities determined via ddPCR were retrieved from clinical notes. The data cut-off date for survival analysis was February 28, 2017.

### Statistical analysis

Data are reported as frequencies, medians (with ranges), or means (±SD) where appropriate. Demographics and clinical characteristics of the patient subgroups within cohort B were compared using chi-square tests, while mutant copy number and treatment response were compared using Mann-Whitney U tests. Iteration method analyses with 5 mutant copies/mL plasma increments were used to generate two strata for survival analysis. Kaplan-Meier analysis was used to evaluate PFS and OS, and a log-rank test was performed to examine differences between the high and low mutant groups in cohort B. Statistical significance was defined by *p*-values less than 0.05. All tests were two-sided. Statistical analyses were performed using R Studio, Version 1.0.136 (RSTUDIO, MA, USA).
